# Melatonin and Female Hormone Secretion in Postmenopausal Overweight Women

**DOI:** 10.3390/ijms16011030

**Published:** 2015-01-05

**Authors:** Ewa Walecka-Kapica, Jan Chojnacki, Agnieszka Stępień, Patrycja Wachowska-Kelly, Grażyna Klupińska, Cezary Chojnacki

**Affiliations:** Department of Clinical Nutrition and Gastroenterological Diagnostics, Medical University, 1 Haller’s Square, 90-647 Lodz, Poland; E-Mails: jan.chojnacki@umed.lodz.pl (J.C.); agnieszka.stepien@umed.lodz.pl (A.S.); wachowskapatrycja@gmail.com (P.W.-K.); grazyna.klupinska@umed.lodz.pl (G.K.); cezary.chojnacki@umed.lodz.pl (C.C.)

**Keywords:** menopause, obesity, melatonin, 6-sulfatoxymelatonin, estradiol, follicle-stimulating hormone

## Abstract

Estrogen deficiency is considered to be the main cause of increased appetite and increased weight in postmenopausal women. In this period, reduced secretion of melatonin (MEL) was also observed. The aim of the study was to evaluate the secretion of melatonin, 17-β estradiol and follicle-stimulating hormone (FSH) in relation to body mass index (BMI) in pre- and postmenopausal women. The study included 90 women divided into three equal groups: group I (control)—women without menstrual disorders, group II—postmenopausal women without change in appetite and body weight, group III—postmenopausal women experiencing increased appetite and weight gain. In each patient, serum melatonin, 17-β-estradiol, FSH and urine a 6-sulfatoxymelatonin (aMT6s) were determined. Compared to the control group, the level of melatonin and estradiol was statistically lower. The FSH level was higher than in the groups of postmenopausal women. No significant correlation was found in all groups between the level of melatonin and the levels of estradiol and FSH. A negative correlation was found between aMT6s excretion and BMI, and a positive correlation between the level of FSH and BMI, mainly in overweight women. The obtained results indicate a significant effect of melatonin deficiency on the process of weight gain in postmenopausal women and justify its use in treatment of these disorders.

## 1. Introduction

Postmenopausal women often have problems with emotions associated with change in appetite [1,2,3]. Increased appetite leads to obesity [4,5,6], which is an important medical and social problem [7,8,9].

The decrease of estrogen production is believed to be the main cause of appetite stimulation. Ovariectomized experimental animals demonstrated rapid weight gain, which can be prevented by estradiol supplementation [10,11]. It is considered that estradiol exerts anorexic effect by stimulating the production of serotonin in the Central Nervous System (CNS) [12]. Estrogens also increase satiety center activity via cholecystokinin receptors [13,14,15]. Moreover, they impair gastric motility [16,17], suppress gastric acid secretion [18] and stimulate the secretion of bicarbonates in the duodenum [19]. Thus, it is reasonable to apply hormone replacement therapy in the prevention of obesity [20]. However, the use of female sex hormones has many limitations due to adverse reactions, including as serious ones as predisposition to blood clots and embolism, myocardial infarctions, strokes or tumors [21,22,23,24,25]. Furthermore, long-term use of estrogens and gestagens tends to increase weight gain [26,27]. Recommended diets do not suppress the appetite, and they are not sufficiently effective. Various dietary supplements are also recommended but strong pharmacological agents “hidden” from the patients may be found in their composition. In recent years, the FDA has drawn up a “blacklist” of dozens of dietary supplements that besides herbal ingredients contain drugs such as sibutramine, furosemide, bumetanide, rimonabant or phenytoin [28]. These potent agents used in an uncontrolled manner can cause very serious side effects.

Some of them (rimonabant) are not approved for marketing in the US and others have been deleted from the list of drugs (sibutramine) because of the risk of severe cardiovascular events. There is an ongoing search for new ways to treat eating disorders, especially in postmenopausal women. Attention has been paid, among others, to the role of melatonin in the process of nutrition. In their first studies, Brambilla *et al.* [29] found that melatonin secretion was higher both in women with anorexia nervosa and in those with obesity than in the control group. At the same time, they noticed in both diseases that melatonin circadian rhythms were disrupted but these changes did not correlate with the degree of body weight deficiency or obesity.

In subsequent years, Ferrari *et al.* [30] observed similar alterations in the rhythm of melatonin secretion in women with anorexia nervosa and simple obesity. They found an inverse correlation between the level of melatonin and luteinizing hormone (LH) in both groups and concluded that the increased secretion of melatonin during the daytime inhibited the gonadal function and played an important role in the pathogenesis of eating disorders.

In turn, experimental studies on animals showed increased resistance to insulin, the development of glucose intolerance and weight gain in pinealectomized rats [31,32]. It was also shown that melatonin supplementation had a beneficial effect on the secretion of leptin, adiponectin and on the level of glucose, cholesterol and triglycerides in animals with induced obesity [33,34,35,36,37,38].

In humans, melatonin (MEL) is mainly applied to improve the quality of sleep, but its usefulness in the regulation of the appetite is also suggested [39,40,41]. Melatonin supplementation is justified in cases of its deficiency [42] which happens, among others, in postmenopausal women [43]. It is also suggested that there is not only time but also cause-effect dependence between melatonin and estrogen secretion. However, the involvement of both hormones in the development of eating disorders and obesity is not sufficiently recognized, which justifies further research.

The aim of our study was to evaluate the secretion of melatonin, 17-beta-estradiol and follicle-stimulating hormone in relation to the nutritional status of postmenopausal women.

## 2. Results

**Figure 1:**
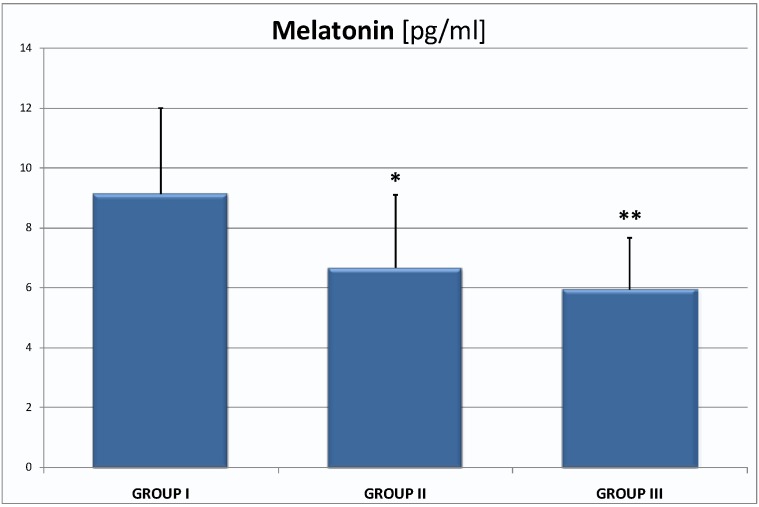
The serum melatonin levels in group I—9.13 ± 2.88 pg/mL, group II—6.67 ± 2.43 pg/mL, group III—5.94 ± 1.73 pg/mL; they are the significant differences in mean value between group I and II; group I and III; * *p* < 0.05, ** *p* < 0.01.

The serum melatonin levels assayed at 9.00 a.m. were in group I 9.13 ± 2.88 pg/mL, in group II 6.67 ± 2.43 pg/mL (*p* < 0.05) and in group III 5.94 ± 1.73 pg/mL (*p* < 0.01; Figure 1).

**Figure 2:**
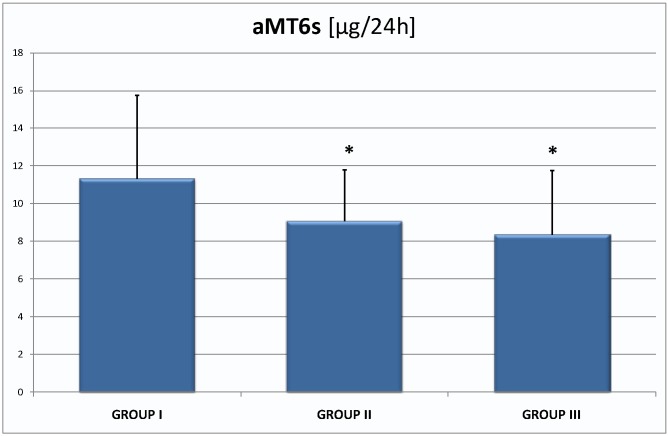
Urinary 6-sulphatoxymelatonin levels in group I 11.32 ± 4.42 µg/24 h, group II 9.08 ± 2.70 µg/24 h and III 8.36 ± 3.39 µg/24 h; they are the significant differences in mean value between group I and II; group I and III; * *p* < 0.05.

Similar differences were found in the results of urinary 6-sulphatoxymelatonin excretion: 11.32 ± 4.42 µg/24 h, 9.08 ± 2.70 µg/24 h (*p* < 0.05), 8.36 ± 3.39 µg/24 h (*p* < 0.05; Figure 2) respectively in group I, II and III.

**Figure 3:**
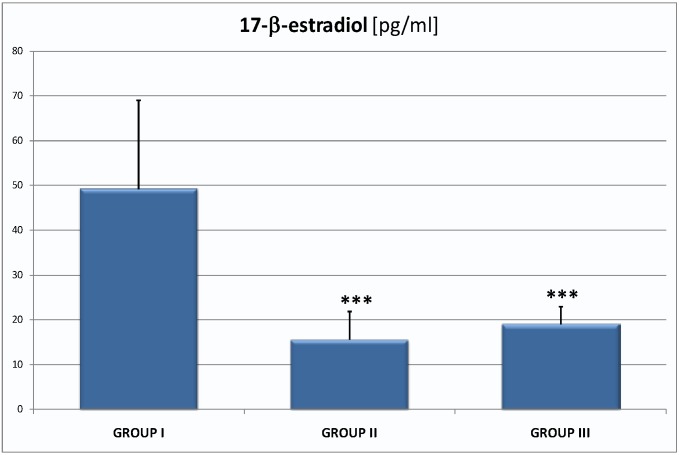
The level of estradiol in group I 49.16 ± 19.80 pg/mL, group II 15.49 ± 6.33 pg/mL, group III 19.01 ± 3.87 pg/mL; they are the significant differences in mean value between group I and II; group I and III; *** *p* < 0.001.

Significantly greater differences were obtained in the results of the tests of female hormones. The level of estradiol was in groups I, II and III respectively: 49.16 ± 19.80 pg/mL, 15.49 ± 6.33 pg/mL (*p* < 0.001) and 19.01 ± 3.87 pg/mL (*p* < 0.001; Figure 3).

**Figure 4:**
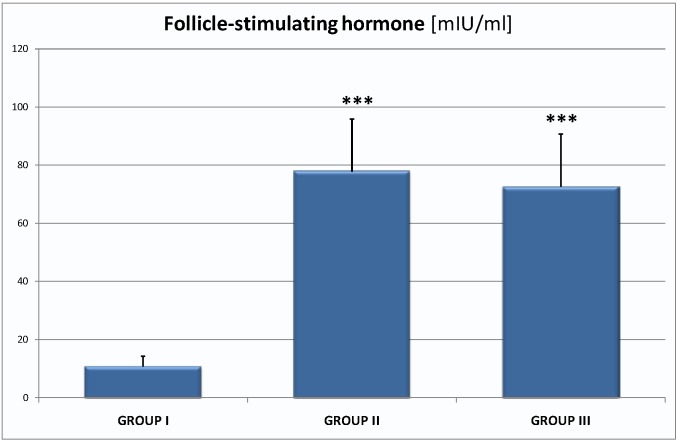
The level of follicle- stimulating hormone (FSH) in the groups: I—10.85 ± 3.40 mIU/mL, II—77.81 ± 18.20 mIU/mL (*p* < 0.001), III—72.63 ± 18.07 mIU/mL; they are the significant differences in mean value between group I and II; group I and III; *** *p* < 0.001.

Decreased level of estradiol in postmenopausal women was accompanied by increased follicle-stimulating hormone (FSH) respectively in the groups: I—10.85 ± 3.40 mIU/mL, II—77.81 ± 18.20 mIU/mL (*p* < 0,001), III—72.63 ± 18.07 mIU/mL (*p* < 0,001; Figure 4).

In these groups a negative correlation was also detected between urinary aMT6s excretion and the level of FSH respectively *r* = −0.5905 and *r* = −0.6423.

The correlations between serum melatonin levels and urinary aMT6s excretion with each other and with appetite were investigated. The significant correlation was found only between serum MEL level and urinary aMT6s (*r* = 0.4599; *p* < 0.05) and between aMT6s and FSH (*r* = −0.7235; *p* < 0.001).

In all groups, no significant correlation was observed between the level of melatonin and body mass index (BMI) (Table 1).

**Table 1:** Correlation between body mass index (BMI) and melatonin, 17-β-estradiol, follicle-stimulating hormone (FSH), 6-sulfatoxymelatonin (aMT6s). *** *p* < 0.001.

Correlation	Correlation Coefficient *r*		
	Group I	Group II	Group III
Melatonin/BMI	−0.3319	−0.3158	−0.2829
17-β-estradiol/BMI	0.3232	0.3682	0.4206
FSH/BMI	−0.1567	−0.2478	0.7487 ***
aMT6s/BMI	−0.3774	−0.4564	−0.8272 ***

Similarly, the correlation between the level of estradiol and BMI was not statistically significant (Table 1).

**Figure 5:**
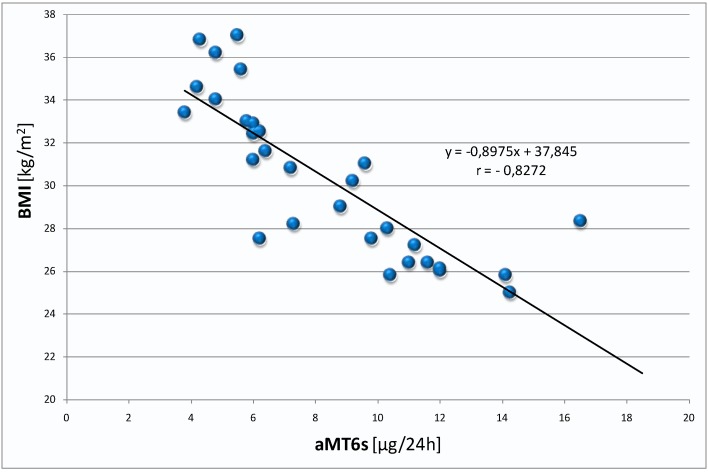
Correlation between urinary 6-sulphatoxymelatonin levels and body mass index (BMI) in the group of overweight women (group III).

**Figure 6:**
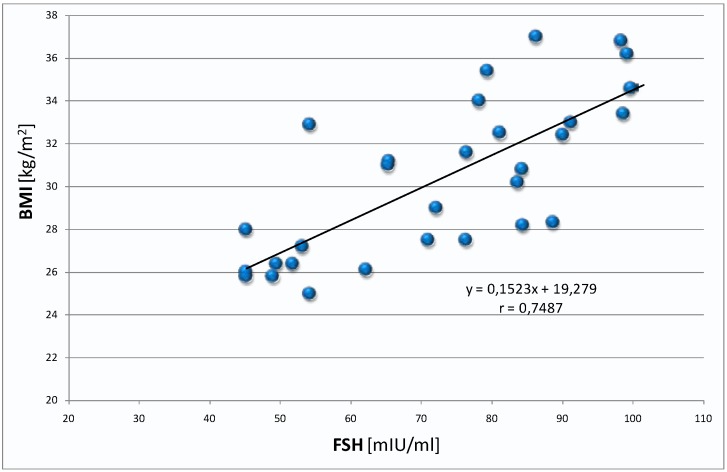
Correlation between the level of FSH and BMI in the group of overweight women (group III).

However, the results of aMT6s and BMI demonstrated a negative correlation, particularly in overweight women: group III (Table 1; Figure 5). Moreover, in this group of women a positive correlation was found between the level of FSH and BMI (Table 1; Figure 6).

## 3. Discussion

Melatonin is a molecule of diverse and still insufficiently known properties [44]. Changes in the amount or rhythm of its secretion were observed in many pathological conditions but it is not always known whether they are the cause or the effect. These doubts are related, among others, to the correlation between melatonin secretion and the secretion of female sex hormones.

Bodis *et al.* [45] demonstrated that melatonin decreases estradiol and progesterone secretion and may contribute to the regulation of the menstrual cycle. In turn, Luboshitzky *et al.* [46] found that the 4-month treatment with estradiol reduces urinary aMT6s excretion. Gruber *et al.* [47] observed a negative correlation between serum level of estradiol and urinary aMT6s excretion in women with secondary amenorrhea. Similarly, Okatani *et al.* [48] detected a negative correlation between the night peak of melatonin secretion and the level of estradiol. They also showed that oral intake of estrogens reduces the nocturnal secretion of melatonin.

The above observations indicate that low levels of estrogen after menopause should result in increased secretion of melatonin. The results of our studies do not confirm such a relationship, because the correlation between the level of melatonin and estradiol was not statistically significant. This may result not only from differences between individual women, but also from a considerable variability in melatonin secretion in the morning. The testing of daily 6-sulfatoxymelatonin excretion is of greater diagnostic value and is considered to be an exponent of melatoninergic system activity.

No significant correlation was found between aMT6s excretion and estradiol level, either. However, a negative correlation was detected between aMT6s excretion and serum FSH level. Similar results were obtained by Vakkari *et al.* [49], who observed in postmenopausal women a negative correlation between the level of FSH and urinary melatonin excretion at night. Furthermore, they noticed that reduced melatonin secretion begins immediately after the age of 40 and can initiate menopause. Blaicher *et al.* [50] found in postmenopausal women high excretion of aMT6s in the case of coexisting hyperprolactinemia and depression but decreased one in obese women.

Toffol *et al.* [43] confirmed decreased nocturnal secretion of melatonin in postmenopausal women but found no correlation between its level and the serum estradiol and FSH levels and also the BMI value.

The finding of a negative correlation between BMI and aMT6s excretion and a positive correlation between BMI and FSH in overweight women is of important cognitive value. This confirms earlier results obtained in experimental studies indicating the beneficial effect of melatonin on metabolism [51,52,53,54]. The regulation of these disorders with the use of melatonin resulted in appetite suppression and weight loss [55,56,57].

A lot of researchers point to the correlation between the low level of melatonin and obesity also in humans [58,59,60,61] and to the usefulness of its application for therapeutic purposes [62,63].

The beneficial effect of melatonin on the reduction of body weight may result from its involvement in the regulation of other hormones. Furthermore, experimental studies have shown that melatonin inhibits the secretion of hydrochloric acid and pepsin [63] and stimulates the secretion of bicarbonates in the duodenum [64]. Its deficiency may lead to a drop of pH in the duodenum, stimulation of duodeno-pancreatic axis, stimulation of insulin secretion and increase of appetite. These changes are unfavorable and difficult to control, particularly at night. In our patients, coexisting sleep disorders may promote the phenomenon of “night snacking” and hyperalimentation.

Estrogen deficiency is considered to be the main cause of eating and sleep disorders in postmenopausal women. The results of our studies indicate that melatonin plays no less important role in the regulation of appetite. This justifies the use of melatonin in the prevention and complex treatment of eating disorders in postmenopausal women, particularly in the case of poor tolerance or side effects of hormone therapy.

## 4. Experimental Section

Ninety women were enrolled into the study, minimum 3 years after their menopause, aged 53–63 years and 30 women, in whom clinical examinations excluded hormonal disturbances and any diseases. Patients were recruited from the Menopause Outpatient Clinic and from the Gastroenterological Outpatient Clinic. Three groups were distinguished:

Group I (control, *n* = 30)—healthy women without menstrual disorders.

Group II (*n* = 30)—postmenopausal women without appetite disorders and change in body weight.

Group III (*n* = 30)—postmenopausal obese women experiencing at that time increased appetite by 5–7 points in a 10-point Visual Analog Scale (VAS) scale and BMI gain by minimum 28% of BMI (Table 2). For all patients, the body weight began to increase in the period of menopause.

**Table 2:** General characteristic of women enrolled in the study. Results of the mean value and standard deviations; ** *p* < 0.01.

Feature	Group I	Group II	Group III
Age (years)	32.4 ± 3.1	57.3 ± 2.1	58.0 ± 4.3
BMI (kg/m^2^)	21.7 ±.1.7	21.8 ± 1.8	30.4 ± 3.7 **
HARS (points)	10.5 ±.6.1	19.7 ±.3.5	18.6 ± 3.1
BDI (points)	6.6 ± 2.9	16.8 ± 2.5	17.0 ± 2.1
GFR (mL/min)	104.8 ± 10.2	96.5 ± 7.1	92.0 ± 11.9

In group II and III the study was initiated 3–6 years after the last menstruation.

The clinical examination determined, among others, the level of anxiety using the Hamilton Anxiety Rating Scale (HARS) and the severity of symptoms of depression using the Beck Depression Inventory (BDI) and imaging examinations were performed (endoscopy, abdominal ultrasonography—USG, computed tomography—CT), as well as the following laboratory tests: blood cell count, C-reactive protein (CRP), bilirubin, alanine (ALT) and aspartate (AST) aminotransferase, gamma-glutamyl transpeptidase (GGT), amylase, lipase, urea, creatinine, cholesterol, triglycerides, glycated hemoglobin.

Exclusion criteria: other organic and metabolic diseases, past surgeries, severe anxiety (over 24 scores in HARS) and/or depression (more than 15 points in BDI) according to the German criteria, *Helicobacter pylori* infection, the use of hormone replacement therapy or other pharmacotherapy.

Seven days prior to the evaluations, all medications were withdrawn and the patients remained on the same diet containing trypthophan-rich products. On the day of the study the patients were administered the same liquid diet (Nutridrink-Nutricia) in the amount of 3 × 400 mL, containing 18.9 g carbohydrate, 6.0 g protein, 5.8 g lipid/100 mL, of the total caloric value of 1800 kcal, and 1500 mL of isotonic still water. Blood samples were drawn from the antecubital vein at 09:00 a.m. and serum was frozen at −70 °C.

The group I patients had their blood drawn in the follicular phase. On the same day, the 24 h urine collection was performed and the samples were kept at 4 °C. At the end of 24-hour collection, the volume of urine was measured and the samples were frozen at −70 °C. Serum melatonin and urinary aMT6s concentration were measured by the ELISA (enzyme-linked immunosorbent assay) method applying IBL antibodies (RE-54021 and RE-54031, IBL International GMBH, Hamburg, Germany) and Expert 99 MicroWin 2000 Reader (BMG Labtech, Offenburg, Germany). Seventeen-β-estradiol concentration in serum was measured by the Elisa test (antibodies Ortho–Clinical Diagnostic Inc., Raritan, NJ, USA) and FSH concentration in serum was measured by the Elisa test (antibodies Vitros Products, Ortho-Clinical Diagnostic Inc., Rochester, NY, USA).

The patients qualified for the study gave their written consent. The approval of the Bioethics Committee of the Medical University of Lodz was also obtained (RNN/45/12/KB from 17 January 2012).

### Statistical Analysis

The nonparametric Kruskal-Wallis test was used in the statistical analysis to compare melatonin, 17-β-estradiol, FSH and urinary aMT6s excretion. The Mann–Whitney test was applied for median comparison. The correlation between the above parameters and the BMI was estimated with the Spearman correlation and linear regression equation.

STATISTICA 9.1 software (AxAP106E735914191-F license, StatSoft Inc., Krakow, Poland) was used for the calculations.

## 5. Conclusions

Melatonin deficiency plays an important role in the process of weight gain in postmenopausal women.

Melatonin supplementation could have a beneficial effect on the reduction of body weight, particularly in the case of poor tolerance of hormone therapy.
